# The relationship between attitude toward physical activity and weight gain in children and young adolescence

**DOI:** 10.3389/fped.2024.1300613

**Published:** 2024-05-07

**Authors:** Ömer Özer, Osman Uyhan, Erhan Devrilmez, İlkay Orhan, Mert Bilgiç, Alkan Uğurlu, Ekrem Yasin Tabak, Oğuzhan Yüksel, Aydın Şentürk, Ayla Karakullukçu, Nebahat Eler, Kürşat Özcan, Selçuk Akpınar

**Affiliations:** ^1^ Faculty of Sport Science, Department of Physical Education and Sport, Bandırma Onyedi Eylül University, Balıkesir, Türkiye; ^2^ Institute of Health Science, Bandırma Onyedi Eylül University, Balıkesir, Türkiye; ^3^ Faculty of Sport Science, Department of Physical Education and Sport, Karamanoğlu Mehmetbey University, Karaman, Türkiye; ^4^ Faculty of Sport Science, Department of Physical Education and Sport, Akdeniz University, Antalya, Türkiye; ^5^ Faculty of Sport Science, Department of Coaching Education, Akdeniz University, Antalya, Türkiye; ^6^ Institute of Social Science, Karamanoğlu Mehmetbey University, Karaman, Türkiye; ^7^ Faculty of Sport Science, Department of Recreation, Dumlupınar University, Kütahya, Türkiye; ^8^ Faculty of Sport Science, Department of Coaching Education, Dumlupınar University, Kütahya, Türkiye; ^9^ Faculty of Sport Science, Department of Sport Management, Kırıkkale University, Kırkkale, Türkiye; ^10^ School of Physical Education and Sport, Department of Coaching Education, Bülent Ecevit University, Zonguldak, Türkiye; ^11^ Faculty of Sport Science, Department of Physical Education and Sport, Nevşehir Hacı Bektaş Veli University, Nevşehir, Türkiye

**Keywords:** physical activity, weight gain, attitude, children, young adolescence

## Abstract

**Introduction:**

The purpose of this study was to investigate the association between attitudes toward physical activity and weight gain among children and young adolescents with an additional focus on the impact of gender on these attitudes.

**Methods:**

Employing a descriptive survey method, data were systematically gathered via purposive sampling from 11 specific cities in Türkiye, ensuring representation from all seven regions. A total of 3,138 students, aged between 9 and 14 years, participated in this study, with a distribution of 46% girls and 54% boys. To assess the attitudes of children and young adolescents toward physical activity, the Youth Physical Activity Attitude Scale was utilized. Height and body weight measurements were taken to determine the body mass index of participants. SPSS 26.0 software facilitated the statistical analyses, including Pearson correlation analysis to explore relationships between variables. Multivariate Analysis of Variance was employed to evaluate the impact of age, BMI, and gender on attitudes toward physical activity.

**Results:**

Participants classified as normal weight exhibited a more positive attitude towards physical activity compared to their obese and overweight counterparts. Moreover, a significant gender difference emerged, with boys demonstrating significantly higher positive attitudes toward physical activity than girls. However, no significant difference was observed in negative attitudes based on gender. The study also revealed that an escalation in negative attitudes towards physical activity correlated with students being categorized as underweight, overweight, or obese, as opposed to having a normal weight status. Additionally, a statistically significant divergence in both positive and negative attitudes towards physical activity was found based on age. Specifically, the results indicated that students aged 9 and 14 exhibited lower levels of positive attitude when contrasted with their counterparts of different age groups. Conversely, in the domain of negative attitudes, students at the age of 9 scored higher than their peers in other age categories.

**Discussion:**

Attitudes towards physical activity can serve as a convenient indicator and guide for assessing the effectiveness of various practices or interventions aimed at promoting physical activity, with recognition of the significant gender difference in positive attitudes among children and young adolescents.

## 1 Introduction

The global prevalence of obesity has exhibited a rapid escalation, emerging as a pervasive and pressing public health concern, with profound implications for the onset of severe morbidities (1). According to the World Health Organization (WHO), overweight and obesity are characterized by an abnormal or excessive accrual of adipose tissue, posing a substantial disruption to overall health (2). This predicament, manifesting not only in adults but also extending its grip on the pediatric and adolescent populations, has garnered substantial attention (3–5). Childhood obesity, once believed to predominantly affect high-income societies, has now emerged as a global epidemic, swiftly infiltrating nations across the economic spectrum (6). According to the WHO, the prevalence of overweight and obesity among children and young adults aged 5–19 years has exhibited a noteworthy escalation, surging from 4% to 8% over the past four decades (2). Furthermore, the global tally of obese individuals in this age group has undergone an alarming rise, soaring from 11 million to a staggering 124 million (2). This proliferation was corroborated by Abarca-Gómez et al. (7), who reported that the prevalence of overweight or obese children and adolescents aged 5–19 years surged from a mere 4% in 1,975 to an astonishing 18% by 2016. A body of literature reveals a consistent pattern, indicating that children who are overweight or obese are significantly more predisposed to carry excess weight into adulthood when compared to their counterparts with normal weight during childhood (8–13).

Numerous studies and health-related reports from various countries have consistently demonstrated a significant surge in the prevalence of overweight and obesity among children and young adults over the past two decades (14). Research conducted in the United States (15–17), China (1, 18, 19), Denmark (20), England (21, 22), Sweden (23), and Norway (24) has consistently documented a rapid escalation in obesity rates among children and young adults. For instance, a recent study by Ma (19) revealed a substantial increase in the obesity rate among Chinese children and youth aged over 7 years, surging nearly sixfold from 2.1% in 1985 to 12.2% in 2014. Alarmingly, projections indicate that this escalating trend may continue, with the obesity rate potentially reaching 28% by the year 2030.

In Türkiye, the prevalence of obesity among children and young adults aligns with trends observed in other countries. According to data from the Turkish Statistical Institute (TSI) in 2019, the obesity rate among individuals over the age of 15 exhibited a significant increase. In 2008, 12.3% of men and 18.5% of women were classified as obese; however, by 2019, these figures had risen to 17.3% for men and 24.8% for women (25). These findings are corroborated by a recent study investigating the obesity levels of Turkish students, which yielded results consistent with the TSI reports. Demir and Bektaş (26) examined 1201 primary school students and identified that 16.9% of the participants were classified as obese. A key contributing factor to this situation has been attributed to low levels of physical activity participation (5, 27, 28).

Engaging in a physically active lifestyle plays a pivotal role in mitigating the risk of obesity across the spectrum of age groups, spanning from children to adolescents and adults (29). Conversely, a sedentary lifestyle stands as a significant determinant influencing both morbidity and mortality rates (30–32). While the manifold health advantages of regular physical activity, particularly among children and young individuals, are widely recognized, reports underscore a disconcerting global trend—the physical activity levels among school-age children often fall short of recommended guidelines (5). For instance, in the United States, less than 40% of school-age children manage to meet the daily physical activity recommendations (33). In Türkiye, the participation levels in physical activity among school-age children mirror this global pattern. According to the Türkiye Nutrition and Health Survey (34) report, it was revealed that a substantial proportion of children failed to meet recommended physical activity levels. Specifically, 52.7% of 9–11 year-olds and 56.2% of 12–14 year-old adolescents did not attain the prescribed levels of physical activity (35). Studies further demonstrate a decrement in physical activity levels as children progress through different age groups (36).

Fogelholm and Kukkonen-Harjula (37) conducted a comprehensive review encompassing 14 studies centered on the physical activity levels of adults. The findings from these studies yielded inconsistent results regarding the direct impact of participating in physical activity on weight gain. Contrarily, a more pronounced relationship was observed between weight gain and alterations or the persistence of physical activity patterns. In a separate review focusing on children, Molnar and Livingstone (38) explored the connection between physical activity and weight gain. Their analysis indicated that among the seven studies included, four identified a relationship between physical activity and weight gain, while the remaining three did not establish a clear association. Wareham et al. (39) conducted an extensive review delving into the intricate relationship between weight gain and physical activity. Their critical examination of research articles revealed three overarching findings: (1) Physical activity emerged as a pivotal factor in preventing weight gain. However, it was suggested that the relationship between physical activity and weight gain may not have been adequately assessed due to limitations in the measurement methods employed. (2) Less weight gain was positively linked to a sustained commitment to exercise. Any deviations from this pattern warranted further exploration and discussion. (3) Physical activity was identified as a crucial component of an overall healthy lifestyle. In light of these results, it becomes evident that there are lingering uncertainties and opportunities for further discourse in understanding the intricate relationship between physical activity and weight gain (39).

Enhancing our comprehension of the factors that influence physical activity engagement among overweight and obese children and young adults is of paramount importance when considering physical activity as a preventive strategy against obesity and excessive weight gain in these populations (40). According to the Theory of Planned Behavior (41), individuals’ attitudes toward physical activity play a pivotal role in predicting their actual participation in physical activity. Here, “attitude” is defined as a spectrum representing an individual's orientation toward a particular goal or an ongoing evaluative process ranging from positive to negative (42). This theory posits that an individual's attitude toward physical activity reflects their subjective assessment of the activity in which they are engaged. When an individual perceives the benefits of physical activity as outweighing the barriers they face, they are more likely to hold a positive evaluation of physical activity and subsequently increase their participation in it. In essence, it suggests that a favorable attitude, driven by a perceived balance of benefits over obstacles, is a crucial driver of increased physical activity engagement.

Conversely, if an individual's perceived barriers to physical activity outweigh the perceived benefits, it is likely to result in a negative attitude, potentially leading to decreased levels of physical activity participation (43). Numerous studies have indicated that attitude serves as a robust predictor of physical activity engagement among overweight and obese children and young adults. These investigations have consistently demonstrated that a positive attitude not only facilitates a better understanding of the advantages of physical activity but also helps in surmounting barriers to being physically active (44–47). However, there is a paucity of studies exploring the relationship between attitudes toward physical activity and weight gain. Among the limited number of studies addressing this aspect is the research conducted by Deforce et al. (40). That study examined the connection between weight status and attitudes toward physical activity in young adults, encompassing a total of 90 young adults with a mean age of 14.6 (SD = 0.9). The findings indicated that overweight and obese young adults exhibited lower levels of positive attitudes toward physical activity. Nonetheless, it's important to acknowledge some limitations of this study. Firstly, it was conducted with a relatively small sample size (*n* = 90) of young adults from a single medical center in Belgium. Secondly, the sample was purposefully selected from a single center, potentially limiting the generalizability of the study's findings.

The distribution and spread of physical activity may vary according to the gender factor (17). Naturally, this situation may lead to different attitudes towards physical activity for both female and male. Similar variations in these attitudes can also be observed in different age groups. Examining 1,129 individuals aged 12–58; Araújo and Dosil investigated how attitudes influence behavioral intentions for physical activity. Results indicated that younger males without higher education exhibit more favorable attitudes towards physical activity and sports compared to females (48). In another investigation involving middle school student, it was found that males exhibited a greater inclination towards engaging in physical activity compared to their female counterparts in terms of willingness to participate. Concurrently, the research results revealed a correlation between individuals' attitudes toward physical activity and their attitudes toward physical education classes, suggesting a parallel alignment (49). In contrast to those results, Zeng et al. found similar and positive attitudes towards physical education activities for girls and boys (50). The existing body of literature thus indicates diverse findings concerning the connection between gender dynamics and attitudes towards physical activity. Consequently, a deliberate examination of the gender variable's impact on attitudes towards physical activity has been defined as an additional research aim in this current study.

Indeed, the body of research exploring attitudes toward physical activity and their relationship to weight gain is relatively limited, particularly with respect to larger and more representative sample groups. Additionally, there exists a notable deficiency in studies examining this relationship in children aged 9–14 years. Consequently, the principal objective of this study is to investigate the association between attitudes toward physical activity and weight gain among children and young adults in Türkiye. The following hypotheses have been formulated: (a) A more positive attitude toward physical activity in children is correlated with a healthier weight status. (b) As negative attitudes toward physical activity increase, students are more likely to fall into the categories of underweight, overweight, or obese compared to students with normal weight. (c) There exists a correlation between age and attitudes toward physical activity. By conducting this study, we aim to contribute valuable insights into the interplay between attitudes toward physical activity and weight status in children and young adults, potentially shedding light on factors that influence physical activity engagement and its impact on weight.

## 2 Method

The study adhered to ethical protocols. Specifically, the study obtained approval from the ethics committee of a state university with the (Approval number: 05-2023/101), ensuring that the research met ethical standards and guidelines for conducting human research. Furthermore, the study demonstrated a commitment to informed and voluntary participation by collecting consent forms from the parents of the participants.

### 2.1 Research design

The research employed a descriptive survey method, which is particularly suitable when little is known about a specific topic and a timely and efficient data collection approach is required (51). The study encompassed seven distinct regions in Türkiye, namely the Mediterranean, Eastern Anatolia, Aegean, Southern Eastern Anatolia, Central Anatolia, Black Sea, and Marmara, within the 81 cities of the country (52). Data were systematically gathered via purposive sampling from 11 specific cities, specifically Tekirdağ, İstanbul, Kütahya, Antalya, Mersin, Karaman, Ankara, Gaziantep, Elazığ, Hakkari, and Giresun. These cities were selected to ensure representation from all regions of Türkiye. Authors reached out to 3,245 students and total of 3,138 students, comprising 46% girls and 54% boys, aged between 9 and 14 years voluntarily participated in this study. Response rate of participants were 96.7%. Data collection was executed in schools. Participants were measured in regular physical education and sport course time. Paper-pencil method was employed to collect the required data. Additionally, the participants' height and weight measurements were taken in a quiet and controlled environment, with participants wearing no shoes, immediately following the collection of instrument-based data. This meticulous approach ensures the reliability and accuracy of the anthropometric measurements.

### 2.2 Measurements

#### 2.2.1 Youth physical activity attitude scale (YPAAS)

To assess the attitudes of children and young adolescents toward physical activity, the Youth Physical Activity Attitude Scale (YPAAS) was employed. This scale was originally developed by Simonton et al. (53) and subsequently adapted to the Turkish context by Uyhan et al. (54). YPAAS is structured as a 5-point Likert-type scale, with response options ranging from “definitely!” (5) to “no way!” (1). It is designed for use with children and young adolescents aged 9 to 14 years. The YPAAS comprises a total of 12 items categorized into two sub-dimensions: positive attitudes and negative attitudes. The positive attitudes sub-dimension consists of 7 items, such as “Physical activity is a valuable part of my life.” Conversely, the negative attitudes sub-dimension encompasses 5 items, including statements like “I do not like to be physically active.” This scale provides a systematic means of gauging the attitudes of children and young adolescents toward physical activity, shedding light on their perceptions and sentiments regarding this important aspect of their lives.

#### 2.2.2 Anthropometric measurements

Height was determined without shoes on a portable stadiometer (Harpenden Portable Stadiometer, Holtain, Crymych UK). Body weight was measured using a calibrated scale (Seca 770 Model scale; Vogel and Halke, Hamburg, Germany). Body mass index (BMI) was calculated as weight in kg over height in meter squared. Following BMI calculations, percentiles (%) were determined using CDC BMI growth chart. Based on that, participants were grouped as underweight (BMI: <5%), normal weight (BMI: 5%–84%), overweight (BMI: 85%–94%) and obese (BMI: ≥%95) (55, 56).

### 2.3 Data analysis

SPSS 26.0 package software was used for the statistical analyses. Pearson correlation analysis was employed to explore relationships between variables. One-way Multivariate Analysis of Variance (MANOVA) was used to evaluate the impact of one or more independent variables on multiple dependent variables simultaneously. We checked basic assumptions such as normality, homogeneity and multicollinearity before moving to analysis. Normality assumption was controlled by skewness-Kurtosis (univariate normality) and Mardia's test (multivariate normality) values. Skewness-Kurtosis (between +1.5 and −1.5) and Mardia's test (.94 < 1.96 coefficient) values met the required criteria (57). Second assumption was homogeneity which was checked by univariate (Levene's test) and multivariate (Box'M test) values. To meet the homogeneity assumption, values should be non-significant (58). Results of dependent variables showed that homogeneity assumption met the criteria. Lastly, multicollinearity assumption was checked by VIF (violence inflation factor) and tolerance values. Results showed that criteria for VIF (1.17 < 10.0) and tolerance (.69 > .40) were met (59, 60). All effect sizes were determined by partial eta squared (*η*2). It was evaluated as ≤0.01 small effect; ≤.0.06 middle effect; and ≤0.14 large effect (57).

## 3 Results

Table 1 indicated means, standard deviations and correlation values for body weight and physical activity attitude variables. Table 1 showed that normal weight students had higher positive PA attitude (*M* = 4.26, SD = 0.57) and lower negative PA attitude (*M* = 1.81, SD = 0.76) than underweight, overweight and obese students. Since higher scores for positive and lower scores for negative indicate better PA attitudes, normal weight students had higher PA attitude than those in other weight categories. Pearson correlation results demonstrated that there was moderately significant correlation between BMI and positive PA attitude (*p *= −.37, *p* < .01). BMI and negative PA attitudes had a moderate significant relation (*r *= .31, *p* < .01).

**Table 1:** Correlation between the body weight and physical activity attitude.

Variables			Physical activity attitude						* *	
		Positive attitude					Negative attitude			
	*N*	*M*	*SD*	*r*	*p*		*M*	*SD*	*r*	*p*
BMI				−.31	.00*				.37	.00*
Normal weight	1,497	4.26	0.57				1.81	0.76		
Underweight	519	3.92	0.70				2.09	0.89		
Overweight	589	4.11	0.61				1.97	0.81		
Obese	533	3.98	0.72				2.24	0.91		
Age				.06		.22			.22	.00*
9	488	3.97	0.77				2.30	0.88		
10	529	4.22	0.59				1.83	0.75		
11	513	4.27	0.59				1.79	0.78		
12	497	4.21	0.58				1.88	0.81		
13	547	4.17	0.62				1.86	0.77		
14	564	4.06	0.64				1.98	0.85		
Total	3,138									

* Correlation significance level *p* < .01.

**Table 2:** MANOVA results for gender, age and BMI.

		*F*	Hypothesis *df*	Error *df*	*p*	*η^2^*
Gender	Wilks’ lambda	5.87	2.00	2,487.00	.00	.01
BMI	Wilks’ lambda	13.05	6.00	4,974.00	.00	.02
Age	Wilks’ lambda	14.59	10.00	4,974.00	.00	.03
Gender × BMI × Age	Wilks’ lambda	2.04	30.00	4,974.00	.00	.01

MANOVA results (Table 2) indicated significant difference by gender [Wilk's Lambda = .99, F_(2, 2487) _= 5.87, *p* < .01, *η*^2 ^= .01] BMI (Wilk's Lambda = .96, F_(6, 5002) _= 16.44, *p* < .01, *η*^2 ^= .02), age [Wilk's Lambda = .95, F_(10, 5002) _= 14.48, *p* < .01, *η*^2 ^= .03], as well as gender by age by BMI [Wilk's Lambda = .98, F_(30, 4976) _= 2.04, *p* < .01, *η*^2 ^= .01].

Univariate analysis represented in Table 3 showed that attitude in terms of the gender variable had a significant effect on positive attitude [F(_1, 3093_) = 6.61, *p* < .01, *η  *^2 ^= .01], not for negative attitude [F(_1, 3093_) = .44, *p* > .01, *η*^2 ^= .00]. Bonferonni follow-up test indicated that boys had significantly higher positive attitude than girls. There was no difference on negative attitude in terms of gender variable. BMI variable had significant difference on both positive [F(_3, 3093_) = 22.38, *p* < .01, *η*^2 ^= .03] and negative [F(_3, 3093_) = 24.04, *p* < .01, *η*^2 ^= .03] PA attitudes. Bonferroni follow-up test showed that normal weight students had significantly higher positive PA attitude than underweight, over weight and obese students. Moreover, over weight students had higher positive PA attitude than underweight and obese students. There is no statistically significant difference between underweight and obese students in terms of positive PA attitude. In univariate analysis of age variable, there was a statistically significant difference on positive [F(_5, 3093_) = 16.17, *p* < .01, *η*^2 ^= .03] and negative [F(_5, 3093_) = 26.38, *p* < .01, *η*^2 ^= .05] PA attitudes. Follow up results indicated that students with 9th and 14th ages had statistically lower positive PA attitude than students with 10th, 11th, 12th and 13th. There is no difference between students with 9th and 14th ages. In negative PA attitude, students with 9th ages had higher scores than all other students.

**Table 3:** Univariate analysis result for gender, age and BMI.

	Dependent variables	*df*	*M^2^*	*F*	*p*	*η^2^*
Gender	Positive attitude	1	2.42	6.61	.01	.01
	Negative attitude	1	.27	.44	.56	.00
BMI	Positive attitude	3	8.31	22.38	.00	.03
	Negative attitude	3	14.64	24.04	.00	.03
Age	Positive attitude	5	6.01	16.17	.00	.03
	Negative attitude	5	16.07	26.38	.00	.05
Error	Positive attitude	3,093	.37			
	Negative attitude	3,093	.61			
Total	Positive attitude	3,138				
	Negative attitude	3,138				

## 4 Discussion

The escalating incidence of childhood obesity has emerged as a significant global public health issue (14). The prevalence of overweight or obese children and adolescents aged 5–19 years, has surged from 4% in 1975 to an alarming 18% in 2016 (7). To mitigate or forestall obesity, precautionary measures, such as fostering a positive disposition toward physical activity, should be implemented for children and adolescents (46). In this context, the primary objective of this investigation was to scrutinize the correlation between attitudes towards physical activity and weight gain among students in elementary and middle schools.

Childhood and adolescent obesity have been a pressing global concern for over two decades, as evidenced by numerous studies (1, 15–19, 25). Research has consistently demonstrated a robust association between the level of physical activity and obesity (5, 27, 28, 61). Furthermore, the attitude towards physical activity is a crucial consideration, as per the Theory of Planned Behavior (41), which posits that individuals’ attitudes toward physical activity significantly predict their engagement in such activities (53).

The initial hypothesis of this investigation posited that children exhibiting a more positive attitude towards physical activity would demonstrate a healthier weight status. Our study's results provided empirical support for this hypothesis, as individuals with a markedly positive attitude were found to maintain a normal weight, contrasting with those holding a less positive attitude. It is noteworthy that only a limited number of studies have thus far delved into the nexus between attitude towards physical activity and weight management. Among these, the study conducted by Deforce et al. (40) merits mention, as it examined the interplay between weight status in young adolescents and their attitudes toward physical activity. Remarkably, this study yielded congruent findings with our own, demonstrating that participants classified as normal weight exhibited a more favorable attitude towards physical activity when compared to their obese and overweight counterparts.

Research dedicated to exploring attitudes towards physical activity has suggested that a negative attitude tends to be a more potent predictor of physical inactivity than a positive attitude (62). Our second hypothesis postulated that an escalation in negative attitudes towards physical activity would correlate with students being categorized as underweight, overweight, or obese, as opposed to having a normal weight status. Our study's outcomes substantiated this hypothesis, revealing that students characterized by underweight, overweight, or obesity exhibited more pronounced negative attitudes towards physical activity when compared to their peers with a normal weight status. These findings are in concurrence with the observations made by Deforce et al. (40), who also noted that negative attitudes towards physical activity were prevalent among overweight and obese adolescents.

The third and final hypothesis of this investigation posited that a relationship existed between age and attitudes towards physical activity. Our research outcomes elucidated a statistically significant divergence in both positive and negative attitudes towards physical activity based on age. Specifically, the results indicated that students aged 9 and 14 exhibited lower levels of positive attitude when contrasted with their counterparts of different age groups. Conversely, in the domain of negative attitudes, students at the age of 9 scored higher than their peers in other age categories. An analogous study, albeit with a different focus, conducted by Mercier et al. (46), examined variations in attitudes towards physical activity with respect to grade levels. Their findings revealed that 6th-grade students exhibited a more favorable positive attitude and a less pronounced negative attitude compared to 7th and 8th-grade students. These outcomes corroborate our own results, suggesting that students aged 10 or 11 (typical ages for 6th-grade students) exhibited the highest levels of positive attitude and the lowest levels of negative attitude towards physical activity.

In the existing body of scientific literature, multiple studies have consistently highlighted a significant correlation between attitudes towards physical activity and the actual level of physical activity engagement (62, 63). For instance, Mercier et al. (46) conducted a comprehensive examination of the interplay between attitudes towards physical education, attitudes towards physical activity, and the intention and behavior concerning physical activity. Their findings robustly underscored a strong positive correlation between a favorable attitude towards physical activity and the self-reported physical activity levels among middle school students. Furthermore, attitudes towards physical activity have demonstrated substantial associations with diverse variables, including social problem-solving skills (64). Within an educational context, it is evident that physical education classes play a central role in striving to ensure that children and adolescents meet recommended levels of physical activity. Consequently, school-based physical education programs have been consistently linked to a positive promotion of physical activity (65). Uddin et al. (66) further substantiated the positive association between attending physical education classes and participation in physical activity, regardless of gender and age. Their study revealed that adolescents engaging in physical education classes for three or more days per week had twice the likelihood of achieving recommended activity levels compared to those not partaking in such classes. In a similar vein, Gao et al. (67) noted that normal-weight middle school students exhibited higher levels of moderate-to-vigorous physical activity during physical education classes when compared to their overweight and obese peers. Consequently, it is conceivable that physical education teachers, along with educational leaders and policymakers, should carefully consider these findings and formulate strategies to provide enhanced opportunities for physical activity to cater to the diverse needs of children and adolescents. Considering these observed correlations and recommendations, it becomes evident that attitudes towards physical activity can serve as a convenient indicator and guide for assessing the effectiveness of various practices or interventions aimed at promoting physical activity.

Although the primary emphasis of this research does not center on the gender variable, an assessment of gender difference revealed that boys had higher positive attitude than girls. Previously reported findings demonstrated that, similarly, males exhibit more positive attitudes toward physical activity compared to females (48, 49). On the contrary, Zeng et al. (50) found that both boys and girls typically demonstrate comparable and positive attitudes toward physical education activities. In our study, gender variable had no effect on negative attitude. Few studies in literature have reported different findings. For instance, Mercier et al. (46) found significant difference on negative attitude in terms of gender variable. It's important to recognize that negative feelings about physical activity are complicated and can be influenced by a mix of personal, social, and environmental factors. More research is needed to understand the detailed aspects of how gender affects these negative attitudes. This could involve refining measurement instruments, considering additional contextual variables, or exploring longitudinal perspectives to better capture the complexity of this phenomenon.

This study has made valuable contributions to the existing body of literature in two distinct ways. Firstly, it underscored the significance of assessing attitudes towards physical activity as a factor of equal importance to the actual level of physical activity among middle school-aged students. The research findings revealed that middle school students exhibited a notably positive attitude towards physical activity coupled with a low inclination towards negative attitudes. Secondly, this study addressed a noteworthy gap in the literature by investigating the relationship between attitudes towards physical activity and weight gain, a relationship that has received relatively limited attention in previous research. As the literature suggests, weight gain among middle school-aged students is a critical concern, and this study has taken a commendable step in examining potential variables that may influence weight gain in this demographic.

While this study made significant contributions to the understanding of attitudes toward physical activity, it is essential to acknowledge certain limitations. Firstly, despite the statistical significance of correlation findings, it is noteworthy that the effect size values were relatively small. This indicates that while relationships between variables were detected, the magnitude of these associations may be limited. Secondly, the study focused exclusively on individuals aged between 9 and 14 years. To broaden the scope of research in this field, future studies should extend their examination to encompass young adolescents aged beyond 15 years and adults. This expansion in age range would facilitate a more comprehensive understanding of how attitudes toward physical activity may evolve across different life stages.

## Data Availability

The raw data supporting the conclusions of this article will be made available by the authors, without undue reservation.
